# Neutrophils: a subgroup of neglected immune cells in ALS

**DOI:** 10.3389/fimmu.2023.1246768

**Published:** 2023-08-16

**Authors:** Wen Cao, Dongsheng Fan

**Affiliations:** ^1^ Department of Neurology, Peking University Third Hospital, Beijing, China; ^2^ Beijing Key Laboratory of Biomarker and Translational Research in Neurodegenerative Disorders, Beijing, China; ^3^ Key Laboratory for Neuroscience, National Health Commission/Ministry of Education, Peking University, Beijing, China

**Keywords:** neutrophils, innate immunity, degranulation, MPO, ALS

## Abstract

Amyotrophic lateral sclerosis (ALS) is a chronic, progressive neurodegenerative disease characterized by the loss of motor neurons. Dysregulated peripheral immunity has been identified as a hallmark of ALS. Neutrophils, as the front-line responders of innate immunity, contribute to host defense through pathogen clearance. However, they can concurrently play a detrimental role in chronic inflammation. With the unveiling of novel functions of neutrophils in neurodegenerative diseases, it becomes essential to review our current understanding of neutrophils and to recognize the gap in our knowledge about their role in ALS. Thus, a detailed comprehension of the biological processes underlying neutrophil-induced pathogenesis in ALS may assist in identifying potential cell-based therapeutic strategies to delay disease progression.

## 1 Introduction

Amyotrophic lateral sclerosis (ALS) is a fatal neurodegenerative disease characterized by progressive degeneration of upper and lower motor neurons (1). Clinical phenotypes of ALS exhibit heterogeneity, including muscle atrophy, weakness, spasticity and pyramidal syndrome, progressive dysphasia, and dysarthria. The average incidence of ALS is estimated to be 1.5 to 2 per 100,000 person­years, with increased incidence observed worldwide over the past decade (2, 3). Several pathological events, including excitotoxicity resulting from excessive glutamate levels, oxidative stress damage, protein misfolding, disruption of the blood-brain barrier (BBB), and reduced energy metabolism, have been hypothesized to contribute to the neurodegenerative process in ALS. TDP-43 is an RNA-binding protein mainly found in the nucleus and plays an important role in RNA cleavage, transport, translation, and stability (4, 5). 95% of ALS patients exhibit abnormal localization of TDP43 in the cytoplasm, which leads to serious consequences such as miscleavage of RNA in the cytoplasm, decreased translation efficiency, and loss of stability, which may be the main cause in ALS (with the exception of SOD1 mutations) (4, 5). However, the exact pathogenesis of ALS remains unclear to date.

Compelling evidence has revealed dysregulated peripheral immunity as a pathological factor of ALS. Whereas the central nervous system (CNS) has traditionally been considered an immune-privileged tissue due to the selective permeability of the blood-brain barrier (BBB), however, a growing number of studies have revealed functional crosstalk between peripheral immune cells and the CNS (6). The peripheral immune system comprises the innate immune system, with neutrophils as its core components, and the adaptive immune system, with lymphocytes as its core components. Different subpopulations of immune cells display different functions in the progression of ALS. For instance, Tregs have shown a protective role (7), while inflammatory monocytes and CD8^+^ T cells may be destructive (8–10). As the key component of innate immunity, neutrophils act as front-line defenders against pathogens by being stimulated and recruited to affected sites through chemotaxis and engaging in degranulation to eliminate pathogens. However, excessive neutrophil activity can result in significant collateral tissue damage. In ALS, researchers mainly focus on monocytes or T cells previously, while potential effects and mechanisms of neutrophils in the pathogenesis of ALS remain neglected. Modulation of peripheral immune cells has been shown to delay the progression of ALS in mouse models (11, 12), offering a feasible and less invasive intervention strategy than modulation of immune cells in CNS and making peripheral immune cells an attractive therapeutic target in ALS treatment (13–15). For example, pharmacological treatment with masitinib delayed peripheral motor pathway degeneration (axonal pathology, secondary demyelination, and the loss of type 2B myofibers) by preventing mast cell infiltration (11). Garofalo et al. found antibody for the α4 integrin, Natalizumab, prolonged the survival time of ALS mice by blocking the extravasation of immune cells in the central nervous system (12). Thereby, searching for precise immunotherapies targeting specific subsets of peripheral immune cells holds great promise in ameliorating immune-mediated pathological deterioration in ALS, and has great benefits in treating ALS. This review aims to summarize the role of neutrophils in ALS in terms of their origin, activation, and functions in ALS, with a specific emphasis on the impacts of the neutrophil granules on ALS, shedding light on the overlooked contributions of neutrophils in ALS and providing a theoretical basis for potential precise immunotherapy.

## 2 A clinical perspective on the linkage between neutrophil counts and ALS

In recent years, the potential link between circulating neutrophil counts and ALS disease progression has been proposed (**Table 1**). In 2017, Murdock et al. investigated the longitudinal association between changes in peripheral immune markers and ALS disease progression. They found that an early elevation of neutrophils was associated with accelerated disease progression reflected by the change in the Revised Amyotrophic Lateral Sclerosis Functional Rating Scale (ALSFRS-R) (17). However, Cui et al. revealed a different conclusion. They found that neutrophil counts increased gradually over time following ALS diagnosis and were negatively correlated with ALSFRS-R score but didn’t find an association between neutrophil changes and ALS progression (9). In 2020, increased expression of CD16 on the surface of neutrophils was observed, which is considered a marker of oxidative stress and phagocytic activity of neutrophils (25) that is associated with ALS disease progression, indicating a highly-activated state of neutrophils in rapidly progressing ALS (20). In 2021, Murdock et al. conducted a prospective cohort study to explore the association between baseline neutrophil counts and ALS survival. The results showed that higher baseline neutrophil counts are associated with reduced survival in ALS. Furthermore, the effect appeared to be sex-dependent, with a more pronounced hazard ratio in females (19), suggesting a potential role of sex hormones in neutrophil activity. However, evidence from mendelian randomization analysis showed that a genetically determined increased neutrophil count was associated with a reduced risk of ALS (95% CI: 0.858-1.000, *P*: 0.049) (18). The controversial conclusions between mendelian randomization and observational studies may be attributed to two factors: firstly, the effect of neutrophils on ALS in mendelian randomization is weak and could be influenced by confounding biases; secondly, previous observational studies mainly focused on the link between neutrophils and ALS after diagnosis, while mendelian randomization analysis revealed the causal relationships between neutrophils and ALS incidence. These inconsistent findings also suggest that neutrophils may play both detrimental and protective roles for neurons at different stages of diseases. To date, no positive immunosuppressive drugs for ALS patients have been identified in clinical trials (26). More attention should be paid to applying precise immunotherapy in different disease stages and specific subpopulations.

**Table 1:** Previous clinical studies on neutrophil counts and ALS disease progression.

Indicator	Duration	Participant	Outcomes	Conclusions	Reference
Neutrophils	2011.3-2014.5	44 control patients and 90 patients with ALS.	ALSFRS-R	ALS patients exhibited a significant increase in the percentage of neutrophils compared to controls. However, the increase was not correlated with the ALSFRS-R score. NMR was significantly increased in patients with ALS and was correlated with disease progression.	(16)
	2014.6-2016.5	35 controls and 119 participants with ALS	change in ALSFRS-R score	Participants with ALS had increased neutrophil counts compared to healthy controls. Early changes in neutrophil counts had a significant correlation with the changes in the ALSFRS-R.	(17)
	–	Mendelian randomization	–	Increased neutrophil count was suggestive association with reduced ALS risk.	(18)
	2011.6-2019.10	269 patients with ALS	mortality rate	Participants with higher early neutrophil counts had a higher mortality rate compared to those with a lower neutrophil count. This effect was more pronounced in females. ALS participants had increased neutrophil presence in cervical and thoracic spinal cord segments compared with control participants.	(19)
	2017.3-2018.8	23 healthy controls and 48 patients with ALS	ALSFRS-R, bulbar subscore of the ALSFRS-R, change in ALSFRS-R, respiratory function	CD16 expression on neutrophils was associated with greater disease severity and faster rate of disease progression in patients with ALS	(20)
	2015-2020	288 ALS patients	Primary outcome: risk of death after diagnosis of ALS Secondary outcomes: functional status and disease progression rate.	Neutrophils increased over time since diagnosis and were negatively correlated with ALSFRS-R score but not associated with risk of death or the disease progression rate.	(9)
NLR	–	80 patients with ALS and 80 matched controls	ALSFRS-R	NLR was significantly elevated in patients with ALS compared with controls.	(21)
	2012.1-2017.8	194 patients with ALS	ALSFRS-R; survival time	A high baseline NLR was associated with a shorter survival period in patients with ALS.	(22)
	2012.1-2018.12	1030 patients with ALS	ALSFRS-R	Higher NLR in patients with sporadic ALS was associated with faster disease progression rates and shorter survival period.	(23)
	2016.3-2020.1	146 patient with ALS	The rate of disease progression (ΔFS score)	NLR positively correlated with ΔFS values. The ΔFS score progressively increased from the lowest to the highest NLR tertile. The mortality rate of patients with a higher NLR value was twice that of the patients with a lower value.	(24)

The neutrophil-derived metric, neutrophil to lymphocyte ratio (NLR), which reflects the balance of peripheral innate and adaptive immunity, has also been indicated as a promising biomarker for ALS disease progression and prognosis. As early as 2009, low-grade systemic inflammation, characterized by an elevated wide-range C-reactive protein (wrCRP), fibrinogen, erythrocyte sedimentation rate (ESR), and NLR, has been proposed as a hallmark of ALS. This correlation was consistently observed in repeated blood draws over time (21). In 2020, research conducted in South Korea divided ALS patients based on their baseline NLR into three groups and revealed that the patients with a high NLR had faster disease progression and shorter survival (22). These findings were subsequently confirmed by a single-center cohort from China and a multicenter cross-sectional cohort study from Italy (23, 24), suggesting that the balance between innate and adaptive immunity plays an important role in ALS progression.

## 3 The production and release of neutrophils

Neutrophils constitute 50-70% of total leukocytes, making them the most abundant leukocytes in circulation (27). They are polymorphonuclear leukocytes originating from myeloid precursors in the bone marrow (BM), characterized by lobulated or rod-shaped nuclei and large cytoplasm of neutral granules (28). These granules primarily consist of lysosomes containing rich enzymes, such as myeloperoxidase (MPO) and neutrophil elastase (NE), which play crucial roles in the phagocytic functions of neutrophils.

Under physiological conditions, neutrophils are generated and differentiated from stem cells to neutrophil precursors in the BM. Upon stimulation, neutrophil precursors can be mobilized from BM into the circulation (29), which is regulated by the interplay between the C-X-C chemokine receptors (CXCRs) family and C-X-C chemokine ligands (CXCL) family (29).

Neutrophils remain in the BM when their surface receptor CXCR4 binds to CXCL12, which is produced by hematopoietic stem cells and BM stromal cells (BMSC). Granulocyte colony-stimulating factor (G-CSF) serves as a major regulator of neutrophil homeostasis (30, 31). When external stimulation occurs, G-CSF can shift CXCR4 on neutrophils to CXCR2, thereby inducing neutrophil mobilization and release.

As the principal regulator of neutrophil biology, G-CSF, a 19.6kd hematopoietic cytokine, has garnered significant attention in the field of ALS. However, clinical and pre-clinical studies yielded inconsistent conclusions regarding the effect of neutrophils increment. Clinical observations have suggested that higher neutrophil level is associated with accelerated ALS disease progression and higher mortality rate (9, 17, 19, 22). Assuming that G-CSF-induced neutrophilia is detrimental to ALS patients, increased expression of G-CSF would be expected to accelerate the disease progression. However, the results of pre-clinical studies do not support the assumption. Animal studies have demonstrated that G-CSF has neuroprotective effects by suppressing endoplasmic reticulum stress and inhibiting pro-apoptotic proteins (32–34). In SOD1^G93A^ transgenic mice, continuous subcutaneous delivery or CNS-targeted overexpression of *G-CSF* can inhibit the apoptosis of motor neurons (35). The inconsistency may arise from two aspects. First, the effects of G-CSF were complex, with dual roles in both the hematopoietic and nervous systems. Systemic delivery of G-CSF can stimulate the proliferation and differentiation of white blood cells, potentially playing a detrimental role in ALS progression. However, G-CSF receptors are highly expressed in motor neurons (35, 36), and their effect on motor neurons is primarily responsible for their neuroprotective role. As a result, researchers are exploring methods to enhance the central neuroprotective effects of G-CSF while minimizing its peripheral effects. In 2011, Henriques et al. found that intraspinal injection of G-CSF AAV significantly delayed the onset of hindlimb paralysis and prolonged 10% survival in SOD1^G93A^ mice (37), which is improved compared with systemic injections. Second, G-CSF mobilizes neutrophil production but also modifies their activation. It is possible that the action of G-CSF is due to enhancing beneficial/pro-resolution processes in neutrophils (29). Neutrophil infiltration without degranulation may stimulate efferocytosis and repair processes (29).

Although G-CSF treatment has shown potential neurotrophic effects in both pre-clinical and clinical studies of ALS, it is important to consider its significant systemic effects, particularly its impact on neutrophilia. Researchers are currently exploring strategies to mitigate potential peripheral effects of G-CSF by administering it intrathecally in ALS, however, the harmful effects due to its invasive nature should not be ignored.

## 4 Potential functions of neutrophils in ALS

**Figure 1:**
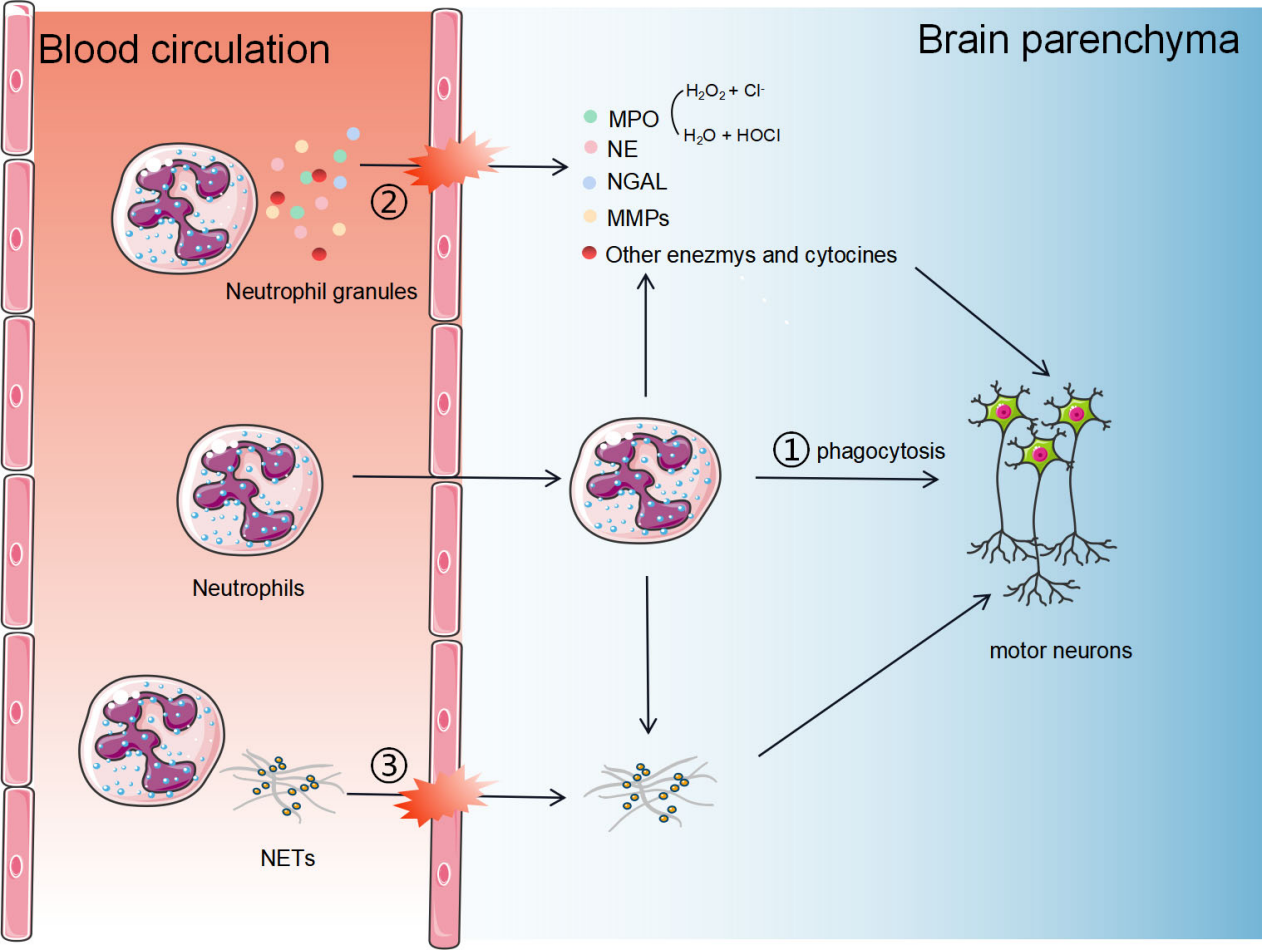
The potential mechanisms of neutrophils in the progression of ALS. Neutrophils contribute to host tissue damage through three major strategies: 1) Phagocytosis. Neutrophils can cross the BBB and directly phagocytose self-components; 2) Degranulation. Neutrophils secrete cytotoxic particles during maturation affecting the immune response. The main components of these particles are enzymes, such as MPO, NE, NGAL, and MMPs; (3) Activated neutrophils can form neutrophil extracellular traps, consisting of chromatin components, MPO and NE attached DNA fibers.

As key effector of innate immunity, neutrophils trigger host tissue damages through three major pathways (**Figure 1**): (1) Phagocytosis: neutrophils can directly phagocytose foreign pathogens and self-components; (2) Degranulation: neutrophils secrete cytotoxic particles during maturation affecting the immune response. The main components of these particles are enzymes, such as MPO, NE, lactoferrin, and metalloproteinases (MMPs) (38); (3) Activated neutrophils can form neutrophil extracellular traps (NETs), which are composed of chromatin components, MPO and NE attached DNA fibers. (4) Neutrophils may crosstalk with other cells, such as microglia and astrocytes. The exact roles of neutrophils playing in ALS pathology and the underlying mechanisms are still unclear. In this section, we describe the three major roles of neutrophils in ALS and propose potential mechanisms by which they contribute to the disease.

### 4.1 Phagocytosis

Whether neutrophils can directly phagocytose healthy motor neurons is not yet clear, while invading to the brain parenchyma is the prerequisite for their phagocytic function. Although it was believed that the BBB provides a privileged immune environment for the brain by blocking peripheral immune infiltration, the current understanding is that the immune privilege in neurodegenerative diseases is relative. Disruption of BBB contributes to the infiltration of peripheral immune cells (39). In disease models of multiple sclerosis (MS) and Alzheimer’s disease (AD), neutrophils have been confirmed to be infiltrated into the CNS (40, 41). Adhesion receptors on endothelial cells, known as integrins, undergo an activation process in response to various stimuli and recruit and promote the attachment of neutrophils to the inflamed endothelium (42). Concurrently, the glycocalyx, a proteoglycan structure, prevents the interaction of surface molecules (43). Once neutrophils adhere to the vasculature, the interaction of endothelial membrane protrusions, containing multiple adhesion molecules, facilitates the migration of immune cells into the brain parenchyma (44). In 2002, Scali et al. reported that peripheral blood neutrophil integrin CD11b was upregulated in AD patients (45). Increased expression of CD11b, which mediates neutrophil adhesion and migration, was positively correlated with AD severity (45). In 2004, Baik et al. showed dynamic imaging of neutrophils entering into brain parenchyma in AD mice, but no such migration was found in wild-type mice (46). In 2015, Zenaro et al. demonstrated that the binding of LFA-1, an integrin expressed on neutrophils, to adhesion molecules expressed on endothelial cells mediated the infiltration of neutrophils into the brain parenchyma (47). Depletion of neutrophils or LFA-1 improved cognitive function and reduced microglia proliferation in AD mice (47). However, whether limiting neutrophil migration and infiltration can delay neurodegeneration in ALS is still unknown.

Figueroa-Romero et al. and Trias et al. showed increasing infiltration of neutrophils with time in ALS mouse models in the spinal cord or the peripheral motor pathway (11, 48). However, previous studies have only demonstrated that neutrophils can be accumulated in the spinal cords of late-stage ALS mice (48) and ALS autopsy (19). It remains unclear whether the accumulation of neutrophils worsens neuronal degeneration or it is a byproduct of neuronal degeneration. There are no studies showing the access of neutrophils into the CNS in ALS mice or patients at the early stage of the disease. Meanwhile, neutrophils are smaller than motor neurons, with a diameter 10 ~12μm. We thus proposed that the neutrophils don’t play a direct phagocytosis role in motor neuron degeneration.

### 4.2 Degranulation of neutrophils

The function of neutrophils relies heavily on the release of cytoplasmic granules. Neutrophils mainly contain three types of cytoplasmic granules. Primary granules, also known as azurophilic granules, are the largest and earliest-formed granules containing proteolytic and bactericidal proteins, including MPO, NE, and arginase-1 (ARG1) (49). Secondary and tertiary granules, also known as specific and gelatinase granules, are enriched with enzymes involved in extracellular matrix (ECM) degradation and regulation, such as the lactoferrin, neutrophil gelatinase-associated lipid transport protein (NGAL) and MMPs (38).

The mobilization of neutrophils depends on the fusion of cytoplasmic granules with the plasma membrane (exocytosis) and endocytose vacuoles (endocytosis). Neutrophil degranulation plays an important role in chronic inflammation (50, 51), which is potentially linked to ALS disease progression.

#### 4.2.1 MPO

MPO is primarily contained in the azurophilic granules of myeloid cells (mainly neutrophils and monocytes), which are among the last to exocytose (52), serving as a specific marker for myeloid cells. MPO can be rapidly released from neutrophils into the circulation in response to the inflammation occurrence. *In vivo*, MPO converts H_2_O_2_ and Cl^-^ to H_2_O and hypochlorous acid (HOCl) (53). The MPO-HOCl system plays a dual role, serving as a defense mechanism against invading pathogens while also potentially causing tissue damage. MPO has attracted considerable attention from researchers in neurodegenerative diseases, including AD, Parkinson’s disease (PD), and multiple system atrophy (MSA) (54, 55). MPO can be detected in both peripheral blood circulation and CSF. In patients with AD and PD, serum MPO is found to be higher than in healthy controls, making it a possible diagnosis biomarker and indicator of therapeutic effects (56, 57). The elevated expression of serum MPO indicates the activation of circulating neutrophils, suggesting the participation of innate immunity in neurodegeneration. In the CNS, highly expressed MPO were observed in both CSF and postmortem brain tissues from patients with AD and PD ^48,49^, while the origin of central MPO is still debated: 1) Studies have suggested that microglia, astrocytes, and neurons in the CNS can release MPO (58, 59), therefore high expression of MPO in the CNS may not solely be attributed to neutrophils; 2)Smyth et al. found that, in brain tissues of AD mice, MPO is highly expressed and originated from neutrophils that infiltrated into the CNS (60). Another research demonstrated that neutrophil-specific MPO-deficient AD mice perform better in spatial learning and memory than controls (61); 3) Another possible mechanism is that the disruption of BBB allows peripheral MPO to invade the brain parenchyma.

To our knowledge, MPO has not been detected in both plasma and CSF in patients with ALS. However, HOCl was elevated in ALS patients, suggesting a potential pathogenic role of MPO in ALS. In SOD1^G93A^ mice, activated MPO/HOCl has been found in motor neurons, and the systemic inhibition of MPO has been shown to inhibit motor neuron apoptosis and ferroptosis and improve motor functions in these mice (62). However, the specific role of neutrophil-derived MPO in ALS is still unclear.

MPO can mediate neurodegeneration through multiple mechanisms: 1) Oxidative stress. HOCl, as the downstream product of MPO, has potent oxidative activity and can induce serious oxidative damage to motor neurons; 2) Disruption of the BBB. MPO-derived oxidants could induce BBB dysfunction *in vitro* and *in vivo (* 63). Other oxidant species produced by MPO, HOSCN, have been shown to reduce the barrier function of cerebral endothelial cells *in vitro (* 64). Disruption of the BBB is an early event in ALS, while BBB hyperpermeability is involved in the late stage of ALS, which has been linked to motor neuron degeneration (65); 3) Induction of inflammatory cytokines release. In addition to its oxidative activity, HOCl can diffuse through the cell membrane and modify proteins (66) and regulate cellular apoptosis (67); 4) Disruption of energy metabolism. MPO/HOCl inhibits intracellular NAD levels, thereby reducing mitochondrial respiration as well as the production of ATP, NAD, and glutathione (68). High concentrations of HOCl can also directly interact with ATP and disrupt energy metabolism (69). 5) Axonal degeneration. HOSCN can act as a switch to trigger necroptosis (70), which also contributes to axonal degeneration in ALS (71).

Due to strong oxidative capacity and cytotoxicity, MPO has emerged as a promising strategy for the treatment of neurodegenerative diseases. Verdiperstat is an MPO inhibitor that has been granted orphan drugs and fast-track designations for the treatment of MSA by the Food and Drug Administration (FDA) and European Medicines Agency. In addition, FDA has approved Verdiperstat in the clinical trials in the HEALEY ALS platform, providing a novel therapeutic option for ALS patients.

#### 4.2.2 NE

NE is a serine protease mainly released from distributed in the azurophilic granules of neutrophils (72), and plays roles as substrates of ECM, ezymogens, adhesion molecules, signaling receptors, cytokines, and so on (52). It removes invading pathogens and inhibits inflammatory responses caused by bacterial infections. At the same time, persistent secretion of NE may lead to tissue damage. In the extracellular environment, NE is capable of cleaving chemokines and cytokines, leading to their activation or inactivation. NE has also been demonstrated to efficiently cleave Aβ1-42 and is closely associated with the inhibition of Aβ1-42 aggregation (73). However, the correlation between NE and ALS remains unclear.

#### 4.2.3 Neutrophil gelatinase-associated lipid transport protein

NGAL, also known as Lipocalin 2 (LCN2), is a 25kd glycoprotein identified as an acute phase protein stored and secreted by neutrophils. Given its stability and the nature of easy detection in the CSF, plasma, and urine, LCN2 is recognized as a suitable diagnostic and prognostic biomarker for neurological disorders such as AD and MS (74–76). Studies have found a high expression of LCN2 in plasma and postmortem brain tissues of ALS patients (77, 78). Additionally, the analysis of ALS patient data from the ALS Knowledge Portal (ALS KP) and Project MinE has led to the identification of 13 genetic variants of *LCN2*, thereby supporting the potential contribution of *LCN2* variants to the pathology of ALS.

Although it’s believed that plasma LCN2 is mainly derived from neutrophils (79), it can also be expressed in other organs and cells, such as kidneys, endothelial cells, liver, smooth muscle cells, and various immune cells (80). In the CNS, LCN2 is mainly expressed in neurons and glial cells, and its expression increases in response to injury or inflammation (81, 82). Recent *in vitro* and *in vivo* studies have reported that LCN2 induces neurodegeneration via several pathways: 1) LCN2 is neurotoxic in ALS. In rodent models of ALS, TDP-43 mutation leads to the release of LCN2 from activated astrocytes. Reduction of LCN2 in astrocytes reduced neuronal death by regulating apoptosis and iron homeostasis (83, 84); 2) Promotion of inflammation. The concentration of LCN2 is correlated with a significant increase in pro-inflammatory cytokines and chemokines both *in vivo* or *in vitro* in a dose-dependent manner (83, 85, 86); 3) Promotion of pro-inflammatory microglial polarization (87, 88).

#### 4.2.4 Matrix metalloproteinases

MMPs represent zinc-dependent proteases mainly produced by neutrophils (89), characterized by digesting components of ECM. MMPs are classified into five subgroups based on their localization and substrate specificity: collagenases, gelatinases, matrix lysins, membrane-type matrix metalloproteinases, and enamel lysins (90). MMP-9, primarily released by neutrophils and macrophages, is among the most extensively studied MMPs in ALS research (91, 92). When activated, MMP-9 hydrolyzes a wide variety of substrates, including ECM proteins (collagen, fibronectin, laminin, thrombospondin, and tendon in C), non-ECM substrates (TNFα, IL-1β, TGFβ, and CXC motif ligands), and neo-substrates (CD36 and citrate synthase).

In MMP-9 polymorphism, the C (-1562) T variant with higher promoter activity in the T allele compared to the C allele has been demonstrated to increase the risk of developing sALS nearly 2.2-fold in the Chinese population (93), suggesting the pathogenic effects of MMP-9 in ALS.

Beuche et al. reported that the levels of MMP-9 were persistently increased in the serum of ALS patients compared to healthy controls. Subsequently, Demestre et al. released that MMP-9 activation in ALS serum occurs prior to the onset of muscular atrophy or peripheral nerve degeneration, suggesting that MMP-9 activation is not merely a byproduct of nerve injury (94). However, the MMP-9 levels in the CSF of ALS patients have been subject to controversy. Two clinical cohorts from Poland showed significantly decreased MMP-9 levels in the CSF of ALS patients (95, 96), while another study reported an elevated level of MMP-9 (97)

MMP-9 appears to play dual roles in ALS. Dewil et al. found that the deletion of *MMP-9* accelerated ALS progression and significantly reduced the survival of SOD1^G93A^ mice, suggesting a protective role of *MMP-9* in ALS (98). However, subsequent research by Lorenzl et al. reported that MMP inhibitor prolonged the SOD1^G93A^ mice survival compared to control (99), suggesting a detrimental effect of MMP-9 on ALS. Mechanically, MMP-9 may play a neuroprotective role in ALS by promoting injured neuron regeneration and elongation via its interaction with the Schwann cell basal lamina, which is essential for creating a passage for sprouting axons. While several studies also proposed the possibility that MMP-9 might aggravate the progression of ALS: 1) Interruption of the BBB integrity. Neutrophil-derived MMP-9 is implicated in exacerbating BBB leakage, inflammatory cytokine infiltration, and brain injury (100); 2) Induction of axonal dieback in fast motor neurons. MMP-9 can break the ECM and disrupt the NMJ structure. Downregulation of MMP-9 in lumbar spinal neurons has been shown to delay axonal dieback and ameliorates motor neuron degeneration in ALS mice (101); 3) Amplification of the inflammatory response. MMP-9 may promote the cleavage of TNF-α and pro-inflammatory cytokines, leading to motor neuron apoptosis (102).

Interestingly, selective neuron death seems to be associated with the *MMP-9* gene in both ALS mice models and patients. Kaplan et al. compared the expression profiles of distinct subpopulations of motor neurons using the microarray and found that the expression of *MMP-9* in fast vulnerable motor neurons is significantly higher than that in the slow motor neurons. Moreover, MMP-9 was reported to be highly expressed in SOD1^G93A^ mice before disease onset, while no MMP-9-positive motor neurons were detected at the end-stage of SOD1^G93A^ mice, suggesting that *MMP-9* is a major pathological factor in ALS progression. In addition, significantly delayed muscle denervation and motor function loss were recorded in genetic ablation of *MMP-9* in SOD1^G93A^ mice, and these mice demonstrated increased survival times compared to the control (103), further implying a substantial role of MMP-9 in ALS progression.

### 4.3 NETs

Highly-active neutrophils can release NETs, which are extracellular, web-like chromatin structures attached with cytosolic granule enzymes and histones on a scaffold of decondensed chromatin (104). The process of NET formation, known as NETosis, is functionally distinct from apoptosis and necrosis. NETs trap, neutralize and eliminate invading pathogens, including bacteria, viruses, and parasites. However, overactivated NETs contribute to the pathogenesis of neurodegenerative diseases.

Recently, NETs have been observed within the cerebral vasculature and parenchyma of AD models and AD patients, suggesting that NETs can potentially harm the BBB integrity and neural cells (47).

Circulating NETs may be a key factor in BBB collapse. During inflammation, Mac-1 and LFA-1, two forms of integrins, mediate the adhesion of neutrophils to vascular ICAM-1 (105, 106). Recent studies have shown that neutrophil adhesion, via Mac-1 or LFA-1, without transmigration, is sufficient to trigger the NET formation and subsequent BBB breakdown (107–109). Zenaro et al. proposed the formation of intravascular NETs as a mechanism of neutrophil-dependent BBB breakdown using two AD models (47). In addition to AD, NETs have also been proposed to contribute to BBB breakdown in stroke models and cerebral malaria (110, 111). Mechanically, intravascular NETs have a direct toxic effect on endothelial cells by releasing proteins such as NE and MPO. NETosis, a process of neutrophil death, further facilitates the release of enzymes mentioned above. NE increases endothelial cell permeability, while MPO and histones induce endothelial cell death (112) and endothelial barrier dysfunction (113). Several lines of evidence indicate that neutrophils can promote vascular damage and BBB breakdown (113).

NETs have also been found in brain parenchyma, which may play a deleterious role in neurons in neuronal diseases (114). NET components can enzymolyze extracellular matrix proteins and activate inflammasome pathways and mitochondrial apoptosis pathways (115, 116). Therefore, the involvement of NETs has been proposed as a novel mechanism for neutrophil-mediated neurotoxicity and neurodegeneration.

Neutrophils in the neuromuscular junction (NMJ) of ALS mice and patients have been found to form NETs, as demonstrated by extracellular web-like fibers of DNA fibers attached with MPO and NE, implying a high cytotoxic potential towards surrounding tissues (11). In the CNS, NETs have been found in the cerebral cortex in AD mice due to the disruption of BBB. However, as of now, no studies have demonstrated the formation of NETs around motor neurons in ALS.

### 4.4 Crosstalking with other cells

In addition to the direct phagocytosis role, neutrophils can interact with multiple cell types in the brain to exert toxic or beneficial effects.

Microglia are the innate immune cells of the brain. Neutrophil-microgial interactions affect microglia reactivity. Neutrophils release inflammatory factors to activate microglia, such as ROS, LCN2, and MMP9 (87, 117). Moxon et al. showed that a decrease in neutrophils reduces the number of microglia and decreases the activation marker CD68 on microglia after cerebral hemorrhage (118). The main component of NETs, LL37, an agonist of the P2X7 receptor, is highly expressed on the cell membrane of microglia which can promote the activation and proliferation of microglia in TBI, ischemic brain injury, and Alzheimer’s disease (119). At the same time, activated microglia produce large amounts of inflammatory cytokines and chemokines that reciprocally promote the recruitment of peripheral neutrophils to the central nervous system (120). In stroke models, reactive microglia engulfed the infiltrated neutrophils around ischemic core (121). Dedepletion of microglia by CSF1R inhibitors increased the number of neutrophils and aggravated ischemic injury (121).

During the inflammatory response, reactive astrocytes can form perivascular scars, thus limiting the spread of neutrophils from damaged to healthy tissues (122). *In vitro*, Xie et al. (123) found that astrocytes inhibited neutrophil apoptosis and degranulation and increased neutrophil phagocytosis and pro-inflammatory cytokine expression (123). Neutrophil-astrocyte interactions also affect astrocyte reactivity. Treatment of mice with anti-Ly6G antibody inhibited astrocyte proliferation (124). In another *in vitro* study, Hooshmand et al. found that neutrophils can induce astrocyte formation by producing C1q and C3a (125).

The above data suggest that neutrophils and astrocytes and microglia are the main sources of cytokines during neuroinflammation and may promote neurodegeneration by interacting with each other to promote the inflammatory cascade response. In the field of ALS, astrocytes and microglia in different stages of the disease play a very different role, in the early stages of the disease the two kinds of cells seem to play protective roles, contribute to the compensatory response of early neuron death, but with the disease progressing, glial cells shit to a neurotoxicity phenotype and cause further deterioration of the disease (126). Whether neutrophils can interact with microglia and astrocytes in ALS and the underlying mechanisms remains largely unknown. From the above studies in other disease models (87, 117), 118, 123), we speculated that neutrophils may aggravate neurodegeneration by activating microglia and astrocytes and translating them into a deleterious phenotype.

## 5 Concluding remarks and future directions

Research on the role of neutrophils in ALS is still in its early stages, and there are many intriguing areas yet to be explored. Firstly, in other neurodegenerative disease animal models, such as AD and PD, neutrophils have been shown to infiltrate into the brain due to the disruption of BBB (47, 127, 128). Although the presence of neutrophils in the spinal cord and NMJ of SOD^G93A^ mice is documented, their ability to cross the BBB at the early stage of ALS remains uninvestigated. Secondly, neutrophil depletion may improve the cognitive function of AD mice (47), while its role in ALS is not yet known. Thirdly, neutrophils exhibit diverse phenotypes, including a subpopulation known as low-density neutrophils (LDN), which has been reported in a variety of disease conditions (129–131). Investigating the potential role of neutrophil subpopulations in ALS is a promising direction. Lastly, ALS is a highly heterogeneous disease, displaying different levels of innate immunity. Establishing an immune stratification based on neutrophils for ALS patients could pave the way for more precise immunotherapy tailored to distinct patient groups. Further research in these areas will advance our understanding of ALS and the contribution of neutrophils to its pathogenesis and progression.

## Author contributions

DF and WC contributed to conception and design of the review. WC wrote the first draft of the manuscript. All authors contributed to manuscript revision, read, and approved the submitted version. All authors contributed to the article and approved the submitted version.
